# On Zebrafish Disease Models and Matters of the Heart

**DOI:** 10.3390/biomedicines7010015

**Published:** 2019-02-28

**Authors:** Panagiota Giardoglou, Dimitris Beis

**Affiliations:** 1 Zebrafish Disease Models Lab, Center for Clinical Experimental Surgery and Translational Research, Biomedical Research Foundation Academy of Athens, 11527 Athens, Greece; tota_giardoglou@yahoo.gr; 2 School of Health Science and Education, Harokopio University, 17676 Athens, Greece

**Keywords:** cardiovascular development, genetic manipulation, phenotype screening, genome-wide association studies, functional analysis

## Abstract

Coronary artery disease (CAD) is the leading form of cardiovascular disease (CVD), which is the primary cause of mortality worldwide. It is a complex disease with genetic and environmental risk factor contributions. Reports in human and mammalian models elucidate age-associated changes in cardiac function. The diverse mechanisms involved in cardiac diseases remain at the center of the research interest to identify novel strategies for prevention and therapy. Zebrafish (*Danio rerio*) have emerged as a valuable vertebrate model to study cardiovascular development over the last few decades. The facile genetic manipulation via forward and reverse genetic approaches combined with noninvasive, high-resolution imaging and phenotype-based screening has provided new insights to molecular pathways that orchestrate cardiac development. Zebrafish can recapitulate human cardiac pathophysiology due to gene and regulatory pathways conservation, similar heart rate and cardiac morphology and function. Thus, generations of zebrafish models utilize the functional analysis of genes involved in CAD, which are derived from large-scale human population analysis. Here, we highlight recent studies conducted on cardiovascular research focusing on the benefits of the combination of genome-wide association studies (GWAS) with functional genomic analysis in zebrafish. We further summarize the knowledge obtained from zebrafish studies that have demonstrated the architecture of the fundamental mechanisms underlying heart development, homeostasis and regeneration at the cellular and molecular levels.

## 1. Introduction

According to the World Health Organization (WHO) global observatory data, 17.9 million people die each year from cardiovascular diseases (CVDs) which is estimated to be 31% of global deaths [1]. CVD involves a spectrum of diseases affecting the heart and blood vessels such as congenital heart disease, stroke, cardiomyopathy, cardiac valve disease and coronary artery disease (CAD) among other conditions. CAD and its main complication myocardial infarction (MI) account for the leading form of cardiovascular disorders and result in the major cause of total human mortality. Over the last few decades, a lot of research studies have been conducted in order to dissect the epidemiology of CAD and further understand the causality and the risk factors underlying its appearance. The traditional environmental factors contributing to the development of CAD consist of established hypertension, elevated blood cholesterol levels, diabetes, obesity, and behavior/lifestyle choices (unhealthy diet, cigarette smoking, physical exercise, harmful alcohol consumption and anxiety/stress) [2].

Nevertheless, population studies are revealing the genetic basis of CAD, highlighting also the complexity of its genetic causality [3]. The development of high-throughput genetic analysis technologies has provided the essential tools for identifying thousands of single nucleotide polymorphisms linked to CAD in large-scale population studies. In particular, twin studies concentrate a range of specific characteristics, which play a pivotal role in the identification of complex trait/disease associations. These studies compare the similarity between identical pairs (monozygotic twins who share the same genetic background) to that of fraternal twins (dizygotic who share about 50% of their genes, like any other sibling) in order to infer a genetic correlation. In addition, they incorporate crucial properties relevant to risk factors such as age (twins have the same date of birth) and lifestyle (similar in a higher degree compared to other family members) as well as allowing gene–gene interactions and epigenetic modification analyses [4]. Therefore, whole-genome and exome sequencing applied in genome-wide association studies (GWAS) and twin/family studies have revealed the genetic variation in a human population as well as 66 causative loci for CAD [5].

Although technological advantages provide cumulative information of genetic causality, the prediction of pathogenicity and the interpretation of clinical consequences of a genetic variant remain challenging. There are several strategies to validate the candidate variants derived from sequencing approaches. Bioinformatic tools provide a powerful advantage to prioritize the candidates by using computational methodologies and algorithms. However, this is largely limited to common variants, as they demand accurate differences in allele frequencies. As a consequence, rare and de novo variants considered as of uncertain significance are categorized between benign and pathogenic [6,7]. Another strategy is observation sharing in order to accumulate exome and genome sequencing data from large-scale studies, which will be broadly available to the scientific community (Genome Aggregation Database, gnomAD). Functional assessment remains the most powerful and well-validated strategy to elucidate the role of a candidate variant to the disease pathophysiology. Nothing is more convincing than in vivo data derived from animal models utilized to characterize the clinical phenotype and the mechanism by which the candidate variants are implicated to the pathogenicity of CAD and other complex diseases. The choice of the right animal is essential to design the most suitable and appropriate approach in order to generate models that will address the aforementioned research questions. The strength and the limitations of each animal model depend on the respective biological system aimed to be studied. Over the last few decades, zebrafish (*Danio rerio*) have provided significant advantages regarding our understanding of cardiovascular disease causality. The biological characteristics, ease of genetic manipulation, facilitation of chemical screening and genetic similarity to humans are only some of the features that contributed to the emergence of this animal model as a useful and valuable tool for the cardiovascular research field. A set of zebrafish models has been generated to study lipid metabolism and hypercholesteremia, which are known major risk factors for development of cardiovascular disease. Zebrafish *ldlr* mutants were validated as a good model to study vascular lipid accumulation, a hallmark of human atherosclerosis [8]. Zebrafish hearts have also revealed age-related changes in cardiac structure and function such as myocyte hypertrophy, ventricular fibrosis and valvular lesions [9,10,11]. Although aging experiments in zebrafish have the obvious disadvantage of long-term planning, since old fish are considered older than two years, these findings suggest that *Danio rerio* could be also used to study age-associated cardiac disease.

In this review, we highlight the importance of zebrafish models for the functional analysis of genes involved in CVDs derived from large-scale human population analysis. We summarize the experimental studies that have been conducted in zebrafish models and how these have augmented the understanding of genes relevant to CAD physiology.

## 2. Fishing for the Right Animal Model

Zebrafish (*Danio rerio*) are a small tropical fish belonging to the minnow family *Cyprinidae* and are native to Southeast Asia. In recent years, zebrafish have emerged as an excellent model system in biomedical research due to its advantageous characteristics. The experimental value of this organism lies in the general biological and physiological features it possesses as well as in the technical and genetic manipulations that can be conducted. Zebrafish are small fish and each breeding pair can produce a large number of offspring weekly. The embryos develop rapidly, exhibiting optical transparency during the first hours post fertilization (hpf) that allow direct observation using light microscopy. Another advantage over mammalian models is the external fertilization and embryonic development, which allows non-invasive techniques to be applied in order to monitor the early developmental stages. Furthermore, zebrafish embryos have the ability to fully function without blood circulation for 4–5 days post fertilization (dpf) by obtaining adequate oxygen through passive diffusion due to their small size. This characteristic gives the advantage to generate and study models of severe developmental cardiovascular disorders that are embryonic lethal in mice [12,13,14,15].

Zebrafish became a popular vertebrate model to study gene function and dissect human genetic diseases. Several research groups have worked on the zebrafish genome sequencing project initiated from the Sanger Institute in 2001 and provided the largest gene set of any vertebrate sequenced [16]. There is high gene conservation which led to the escalated use of zebrafish as an experimental system to model human diseases. Despite its apparent simplicity, the zebrafish heart exhibits similar features to the human heart in terms of physiology including heart rate, contractile dynamics and action potential [17,18,19,20]. Although the zebrafish heart is two-chambered, providing easier imaging capabilities, it shares fundamental properties with humans. Early developmental processes and signaling pathways are conserved between species, and forward-genetic screens in zebrafish have identified critical pathways in cardiovascular diseases that simulate those of higher vertebrates. In addition, some physiological functions are comparable. For example, the heart rate of zebrafish (± 150 bpm at 72 hpf) is closer to humans than mice (>500 bpm) [21]. On the other hand, zebrafish studies also have several obvious limitations when it comes to study septal development, for example, metabolism or blood pressure. In addition, the utility in genome engineering through a broad gene tool box and large-scale drug/chemical/physical compound screening to embryos and larvae, have established zebrafish as a valuable animal model in fundamental research and translational medicine.

## 3. The Pool of Engineering Tools

### 3.1. Genetic Approaches

Nowadays, there is a wide range of strategies aiming to perform genetic manipulation in order to study and deeply understand the regulatory mechanisms of the pathophysiology of complex diseases. The ease of genome engineering and the plethora of genetic tools lie at the core of the zebrafish models for human disease generation. The genetic landscape of zebrafish carries a whole-genome duplication (WGD) that revealed many interesting features when compared to the human genome [22]. It was found that 82% of human morbid genes enlisted in Online Mendelian Inheritance in Man (OMIM) database are related to at least one zebrafish orthologue and after a similar comparison, 72% of zebrafish genes have been identified as orthologues to human genes in related GWA studies [16]. Due to this specific feature, gene functional redundancy needs to be taken under consideration during zebrafish modeling design. A recent reported example is the redundant roles of zebrafish *smyd1a* and *smyd1b* paralogues [23]. At this study, it was shown that both Smyd1a and Smyd1b were localized in skeletal and cardiac muscles and overexpression of *smyd1a* efficiently compensated the loss of *Smyd1b* in mutant zebrafish and rescued the provoked myopathic phenotype. However, *smyd1a* was not transcriptional activated in *smyd1b*-deficient zebrafish, which is a case of functional redundancy but not of genetic compensation. In another recent study, it was demonstrated that the genetic *actc1b* zebrafish mutant exhibits a milder myopathy phenotype due to the compensatory transcriptional upregulation of an actin paralogue [24]. Accumulating evidence supports the notion that genetic compensation could influence the severity of mutants in genetic disease models.

Genetic compensation has been documented in a number of animal models as a mechanism to fine-tune their transcriptome in order to adapt their fitness and maintain their viability caused by genetic changes. There are a lot of studies focusing on the functional and genetic compensation established in model systems, like Arabidopsis [25,26], yeast [27,28], mouse [29,30] and zebrafish [31]. This buffering system can lead to discrepancies regarding the phenotypical outcome of a disease-caused mutation in model systems [32,33,34]. While toxicity and off-target effects caused by knockout reagents could result in these phenotypic differences, a recent study in zebrafish proposed that gene expression profiling and genetic composition attribute to the observed differences [32]. It was shown that most *egfl7* (*epidermal growth factor-like domain, 7*) mutants have no phenotype, while knockdown of *egfl7* leads to severe vascular defects. It was demonstrated that upregulation of other extracellular matrix (ECM) genes (especially Emilins) have occurred in *egfl7* mutants but not in knockdown embryos. Despite its fundamental role in whole organism robustness, the mechanisms that drive genetic compensation remain poorly understood. In a recent review, two models were proposed: (a) the DNA damage response induces chromatin reorganization so as to increase chromatin accessibility to compensatory gene regulators, and (b) the mutated regions produce transcripts that are targeted for degradation and subsequently, the RNA fragments induce a compensatory response either by triggering chromatin remodeling or guiding common (for mutated and compensated genes) RNA binding proteins (RBP) and/or miRNAs to act instead of compensating gene mRNA stabilization [35].

Despite the overall biological limitations, there is a wide toolbox for genetic engineering that facilitates the modeling of human diseases and we will summarize the most commonly used tools to manipulate the zebrafish genome. Forward and reverse approaches have been successfully applied in zebrafish to identify molecular pathways and to elucidate the role of known or novel genes related to human diseases such as CAD. Forward genetics, i.e. by using chemical mutagens like *N*-ethyl-*N*-nitrosourea (ENU) to induce random mutation, is widely used in zebrafish as it can overcome the limitations of the other vertebrate models like suitable breeding scheme, genomic architecture, large-scale husbandry and ease in monitoring early developmental phenotypes [21]. Numerous large-scale genetic screens have identified several mutant phenotypes related to cardiac, vascular, hematopoietic and other developmental systems [36,37,38,39,40,41]. Based on the phenotypical screening, the characterization and identification of the responsible (mutated) genes provided new insights to the pathophysiology of human diseases. Regarding the cardiac screen, more than 50 lines were isolated that led to the unraveling of crucial regulators during cardiac development [42,43]. Although most of these mutations would cause early embryonic lethality when homozygous (as in human embryo development as well), heterozygous carriers could still have an increased risk to develop cardiovascular disease. One of the most severe heart-specific mutant lines is the *silent heart* (*sih*) which causes a non-contractile heart phenotype [43]. The *sih* embryos survive until 7 dpf due to their ability to uptake adequate oxygen though diffusion and so this line serves as an excellent model to study the structural changes and molecular pathways involved in cardiac disorders while this phenotype would be lethal in other vertebrates. The combination of ENU mutagenesis with transgenic lines harboring reporter genes such as *Tg(flk:EGFP)* and screens on specific cardiac development and morphogenesis have also been conducted [36,44]. Another powerful methodology widely used in zebrafish is the targeted gene expression by using the Gal4-UAS system in yeast [45]. This valuable two component system, in combination with stable transgenic zebrafish lines, has successfully utilized specific gene expression, tissue-specific labeling and cell ablation [46,47,48].

In recent years, zebrafish have become an attractive model system due to the successful application of reverse genetic approaches as well. The most commonly applied technique is gene knockdown by injecting morpholino oligomer molecules (or simply “morpholino”) at the one-cell stage zebrafish embryos to investigate if knocking down a gene causes a phenotype. Morpholinos (MO) are chemically modified oligonucleotides with base-pairing ability similar to the natural oligomers and are effective in a dose-dependent manner [49]. The MOs are designed to prevent either the translation by blocking the translational start sites or the splicing by targeting the splicing junctions. Although this technology has been widely used and was a standard approach for a generation of anti-sense knockdown mutations in zebrafish due to their time and cost effectiveness, there are several concerns about off-target effects and p53-induced apoptosis [31,50,51]. The description of the function of a novel, unknown gene relying exclusively on morpholino data should be accompanied by several controls, including multiple morpholino targets etc., as described in [51]. Once validated and properly controlled, MO-induced knockdowns can quickly generate large numbers of morphants, therefore facilitating functional analyses.

Fortunately, recent advances in reverse genetic approaches also revolutionized the toolbox of zebrafish genome engineering. Genome editing with zing finger nucleases (ZFNs), transcription activator-like effector nucleases (TALENs), and clustered, regularly interspaced, short, palindromic repeats/Cas (CRISPR-associated) (CRISPR/Cas) systems were applied for targeted genome modifications [52,53,54,55,56,57,58,59]. CRISPR/Cas has accelerated the systematic efforts of silencing most of the zebrafish protein-coding genes using the well-annotated zebrafish genome sequence, high-throughput sequencing and efficient mutagenesis [60]. An active project of Kettleborough et al. has identified mutations in the orthologues of 3188 of the 5494 genes currently associated with human disease in genome-wide association studies [60], providing a resource to facilitate the identification of candidate genes responsible for inherited diseases. Specifically, a recent published study has effectively used the CRISPR/Cas9 nucleotide editing strategy to model human cardiovascular diseases [61]. In this study, they generated four patient-specific knock-in zebrafish lines carrying distinct human cardiovascular-disorder-causing missense mutations in their zebrafish orthologous genes by introducing small nucleotide changes in the zebrafish genome. The mutated genes (*abcc9*, *kcnj8* and *pln*) encode for subunits of an ATP-sensitive potassium channel (K_ATP_) linked to Cantú syndrome, a rare genetic syndrome included in cardiovascular disorders. As CRISPR/Cas9 methodology evolves, the capacity of this system to generate specific patient-derived mutations in animal models promises a broad application paving the way for new therapeutic strategies [62,63].

### 3.2. Technical Approaches

To improve our understanding on heart formation during development, specific pathways and regulators must be identified as well as their role under diseased conditions. A recently developed strategy focusing on unraveling genes restricted to well-defined regions of the heart to ensure proper function, morphology and shape is the *tomo*-seq technique [64,65]. This method is based on cryosectioning of an embryo or tissue of interest and performing RNA-seq on the collected individual sections. Application of *tomo*-seq in combination with high-throughput RNA sequencing generated a high-resolution genome-wide atlas of gene expression in the regenerating zebrafish heart [66,67]. These two studies revealed over 1100 genes differentially expressed in cardiac sub-compartments. Specifically, the role of bone morphogenetic protein (BMP) signaling in zebrafish cardiomyocyte de-differentiation and proliferation, as well as myocardial regeneration, was identified. In addition, Islet-1 was shown to regulate the spatially-restricted activation of Wnt/β-catenin in pacemaker cells, therefore controlling heart rate. The advantage of this technique relies on the fact that gene expression patterns can be correlated with high spatial resolution. For example, when looking at the gene expression pattern of a heart following myocardial infarction, one can distinguish between genes that are upregulated in distant unaffected areas of the heart from the infracted area or the border zone. This can more precisely identify which genes are the strongest candidates as potential therapeutic targets.

Since the zebrafish has proven to be an excellent model for human cardiac research, another approach to study cardiovascular diseases is the structure of in vitro three-dimensional (3D) heart-like cell aggregates, consisting of myocardial tissue formed spontaneously from enzymatically digested whole embryonic zebrafish larvae (zebrafish heart aggregate(s)—ZFHA(s)) [13,68]. The ZFHAs spontaneously form and become a stable contractile syncytium consisting of cardiac tissue which can be a platform for further analysis of in vitro cardiac maturation, regeneration, tissue engineering and safety pharmacological/toxicology testing. Although mammalian in vitro systems (cell line, organoids) have been widely used, ZFHA can complement cardiac research of healthy and hypertrophic myocardium. In addition, zebrafish hearts can be cultured as explants for several weeks while maintaining their contractility and thus provide an ex vivo tool for studying cardiac regeneration mechanisms [69]. Since zebrafish cardiomyocytes retain their proliferative potential throughout their lifetime, regenerative events can be monitored live and reprogramming pathways are dissected, which is not the case in mammalian in vitro systems of differentiated cardiomyocytes. Finally, the development of MUSCLEMOTION software has been designed to assess contraction in cardiac model systems, including zebrafish hearts [70]. It is an automated open-source software tool aiming to quantify cardiac contraction noninvasively and monitor responses to drugs and diseases.

## 4. Investigation of Human Cardiac Diseases under the Light of Zebrafish Research

**Figure 1:**
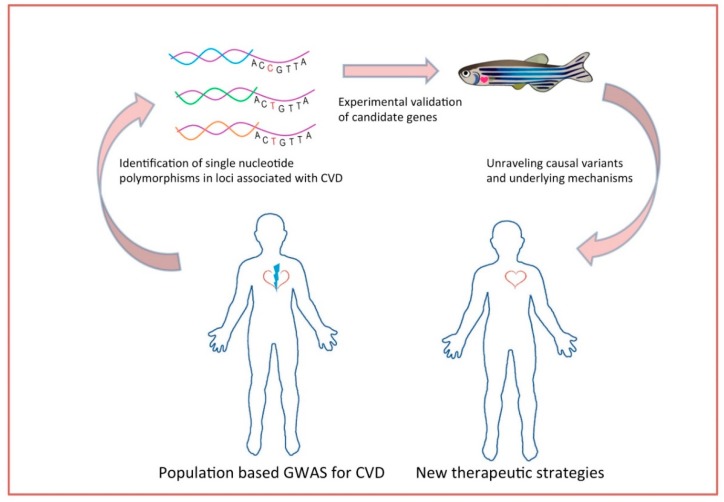
Next generation sequencing and genome-wide association studies (GWAS) studies identify multiple loci and polymorphisms that can be easily studied in vivo in zebrafish. CVD = cardiovascular disease.

Genetic disposition, jointly with traditional risk factors, is considered to manifest in cardiovascular diseases. For many decades, epidemiological studies focused on the effort of unraveling the causes and specific predictors for CVD. Up to now, modifiable and non-modifiable risk factors such as obesity, hypertension, smoking, diabetes, blood pressure, and sex have been studied [71,72,73,74]. It is also known that CVDs have high heredity and that 40–60% susceptibility is attributed to genetic factors [3]. To that basis, validation of candidate genes and elucidation of mechanisms underlying the pathophysiology of cardiovascular diseases provides deeper knowledge towards the development of new therapies. The ability to do high-throughput chemical screens in zebrafish embryos has major translational research implications. A zebrafish model of arrhythmogenic cardiomyopathy was generated expressing a mutation of plakoglobin found in humans. A high-throughput chemical screening of compounds that rescue the phenotype identified a molecule (SB216763) implicating the Wnt signaling pathway in the pathophysiology of the disease [75]. Large scale population-based GWAS have been performed to uncover and identify the genetic variants underlying this heritability. Analysis of genotyping frequencies in such studies unravels genomic regions that harbor determinants of CVDs (and other complex traits). Variants found in GWAS are single-nucleotide polymorphism (SNPs) that are not necessarily located in coding regions and need to be further analyzed and prioritized [76]. GWAS and meta-analysis have identified CAD-associated loci but the mechanism through which these specific variants function remains unclear [5,77,78]. Given the aforementioned advantages of zebrafish and the existing functional toolbox, this animal model provides the platform of validating the candidate causal genes that have arisen from GWAS analyses (Figure 1).

Atrial fibrillation (AF) is the most common sustained arrhythmia and can lead to heart failure and cardiovascular death. Due to the similarity of electrical properties of zebrafish heart to those of the human heart, zebrafish have been established as a powerful animal model to study cardiac arrhythmias [17,18,20,79]. Previous GWAS have unraveled copy number variations (CNVs) in the potassium interacting channel 1 gene (*KCNIP1*) which associated to AF susceptibility. In a recent study, it was demonstrated that *KCNIP1*-knockdown and overexpression in zebrafish hearts modulates atrial rates [80]. Intronic CNV in the *KCNIP1* gene determined the mRNA level of *KCNIP1*, and *KCNIP1*-encoded protein KCHIP1 was linked to the mechanism of AF, maintaining high atrial rates which indicates a possible target for AF treatment [80]. Later on, E637K *KCNH2* mutation (potassium voltage-gated channel subfamily H member 2), which has been identified in long QT syndrome type 2 (LQT2) patients, was evaluated in larval zebrafish [81]. By using an MO silencing strategy, rescue experiments and QT measurements, the severe repolarization phenotype was recapitulated, which highlighted the utility of the LQT2 zebrafish model for functional analysis of *KCNH2* mutation using microscopy and electrophysiology. Another study focused on the transcription factor *PRRX1* as it is a strong candidate gene based on previous genome-wide association studies on AF [82]. In this work, after resequencing the *PRRX1* locus (~158 kb region in AF cases) to identify common and rare SNPs variants, they confirmed that the causative variant resides in an upstream enhancer of *PRRX1* modulated by SNP rs577676, which reduces PRRX1 expression. CRISPR-Cas9-mediated knockout of *PRRX1* in human embryonic stem cells (hESCs) and morpholino-mediated knockdown of the putative *PRRX1* orthologues in the zebrafish (*prrx1a* and *prrx1b*) showed that even a small modulation of *PRRX1* expression may be sufficient to modulate the atrial action potential duration (APD), a hallmark of AF.

GWAS studies on idiopathic cardiomyopathies have implicated polymorphisms in small heat shock protein, beta 7 (HSPB7) as potential contributors and a recently published work highlighted its cardioprotective role [83]. The research group showed that zebrafish *hspb7* mutants (TALEN-generated) display mild focal cardiac fibrosis, cardiomegaly and sarcomeric abnormalities. They also identified large cytoskeletal proteins Filamin C (FLNC) and TITIN as HSPB7 binding partners and proposed that *hspb7* functions through a damage-induced network as loss of *hspb7* stimulated autophagic pathways and inhibition of autophagy (treatment with bafilomycin A (BafA) and chloroquine (CQ) inhibitors) in *hspb7* mutants, resulting in increased sensitivity and more severe cardiac defects. Another group used single-stranded oligodeoxynucleotides to precisely introduce the human PBX3 p.A136V variant in the homologous zebrafish *pbx4* gene through CRISPR-Cas9 genome editing in order to test whether this Pbx gene variant (previously found to be enriched in a congenital heart defect (CHD) patient cohort) acts as a genetic modifier in zebrafish heart development [84]. It was shown that the *pbx4* p.A131V variant enhances myocardial morphogenesis defects caused by loss of the CHD gene, cardiac specification factor, *hand2*. The study provides an example of precision genome editing in zebrafish to demonstrate a function for a human disease-associated variant.

Valvulopathies are diseases of the cardiac valves (mainly mitral and aortic) and non-syndromic mitral valve prolapse (MVP) is a common degenerative valvulopathy that can cause heart failure and sudden death. Studies in zebrafish revealed the effect of intracardiac flow dynamics on their development [85]. A meta-analysis of two GWAS studies focusing on the biological pathways involved in MVP unraveled six loci-residing candidate genes and highlighted the role of LMCD1 and *tensin1* (TNS1) after functional analysis using zebrafish embryos [86]. The zebrafish *lmcd1* morphants exhibit significantly increased atrioventricular regurgitation and moderate reduction in cardiac looping while a similar phenotype was observed for *tensin1*, thus supporting their role as candidate genes for MVP pathogenesis. Atrioventricular septal defects (AVSD) also represent abnormalities in atrioventricular valves, and atrial and ventricular septa. The study by Ferese et al., identified *NFATC1* rare variants in a small but significant proportion of cases from two cohorts of AVSD patients [87]. The authors observed cardiac looping defects and altered atrioventricular canal patterning in the *nfatc1* zebrafish mutants, providing evidence of their functional relevance in vivo and supporting a role of defective NFATC1 function in the etiology of AVSD. Another recent study aimed to functionally characterize the zebrafish orthologues of six human candidate genes (*gng11*, *syt10*, *rgs6*, *hcn4*, *neo1* and *kiaa1755*) in GWAS-derived loci for heart rate variability using a large-scale, image-based screen in zebrafish embryos and larvae [88]. The authors identified nine zebrafish orthologues and after generating CRISPR-Cas9 mutants, the embryos showed sinoatrial pauses and arrests, cardiac edema and uncontrolled atrial contractions as well as abnormalities in cardiac morphology and body size. Following this, they highlighted *HCN4* as a druggable gene using the Drug Gene Interaction Database (DGIdb) and revealed several interaction partners for all the tested causal genes that can be drug-treated based on the current pharmaceutical standards.

GWAS on cardiovascular disorders have focused on identifying traditional risk factor-related loci such as those that are lipid associated. Although mutations resulting in hypercholesterolemia (such as mutations in *LDLR*, *APOB* and *PCSK9*, as well as SNPs at numerous loci) have been reported, only approximately 20% of the variation in low-density lipoprotein cholesterol (LDL-C) levels is explained [89,90,91,92,93]. Due to the fact that isolated populations are enriched in genetic variations that are otherwise rare, studying these populations offers specific advantages [94]. A recently published work conducted an association analysis in the Amish population and unraveled a novel haplotype with elevated LDL-C levels to be correlated with a region containing eight candidate genes [95]. Follow-up functional analysis in a zebrafish model system showed that the overexpression of the transcribed pseudogene, *APOOP1*, increased the LDL-C levels on both the control and those supplemented with a 4% w/w cholesterol (HCD) larval diet and upregulated the expression of genes involved in cholesterol synthesis. In addition, overexpression of *APOOP1* resulted in an approximately 20-fold increase in the average number of vascular plaques and suppression of its parent gene, *apO*, increased LDL-C and plaques suggesting a regulatory interaction between these two genes. Another factor that could eventually increase the risk for CVD is the excessive lipid deposition within adipose tissue (AT). A large-scale meta-analyses of GWAS has identified several loci associated with this trait including Plexin D1 (*PLXND1*)—a gene known to modulate angiogenesis—and Minchin et al., performed a functional analysis to elucidate its role in body fat distribution in a zebrafish system [96]. The group observed that a null mutation in *plxnd1* had a reduced capacity to store lipids in visceral AT (VAT) and that type V collagens were upregulated, suggesting that they mediated the inhibitory effect of Plxnd1 on VAT growth. One more study that used the zebrafish model to validate a rare causal gene involved in congenital cardiomyopathy resulting in a lethal restrictive phenotype was performed by Louw et al. [97]. In this work, whole exome sequencing and linkage analysis was done in a Caucasian family with unaffected and affected (twin) siblings to identify the genetic basis of this novel characterized heart disease. The authors found two variants in the *KIF20A* gene and demonstrated that MO-mediated *kif20a* knockdown zebrafish embryos develop a progressive cardiac phenotype (red blood cells proximal to the atrium, relative tachycardia and cardiac edema), suggesting that *kif20a* is evolutionary conserved in heart development and required for proper heart function. These last studies are examples of how the zebrafish model system facilitates the validation and functional analysis of genes not only derived from large-scale gene association studies, but also can be a platform of studying novel and rare genetic variants that contribute to multifactorial complex diseases (Table 1).

## 5. Zebrafish Heart as an Injury Model

Despite advances in current therapies and preventive medicine for myocardial infarction (MI) and heart failure (HF), cellular and molecular mechanisms underlying their pathophysiology still remain unclear. During HF, there is a progressive loss of cardiomyocytes (CMs), which eventually leads to the formation of fibrotic, non-functional scar development. In order to gain deeper knowledge into human cardiac repair, various injury models have been established. Although newborn mice exhibit a heart regenerative capacity, it is lost by seven days after birth and the adult mammalian hearts retain a low capacity for regeneration, mainly due to the cell-cycle arrest of CMs [98,99,100,101,102,103]. Unlike mammalian models, zebrafish obtain a remarkable ability to replace cardiac tissue after injury and thus provide an ideal model to study the key orchestrators of heart regeneration.

The role of cell-specific contributions and immune responses during heart repair has been extensively studied [104,105]. Among the earliest responses, it is the activation of epicardium and cardiac endothelium and the fast revascularization of the damaged area [106,107]. It has been shown that in the absence of angiogenic sprouting in injured zebrafish hearts, CM proliferation is blocked and hearts fail to regenerate [107]. In addition, a direct macrophage response modulates revascularization as delayed recruitment of the cell population interferes with revascularization [108]. Epicardial cells undergo an endothelial-to-mesenchymal transition in order to invade and infiltrate the wound so as to support a regenerative response (reviewed in [106,109]). A second crucial cell population with a highly significant role upon cardiac injury is fibroblasts, which accumulate at the injury site and form an extra cellular matrix-rich scar. In the cryo-injured zebrafish heart, fibroblasts contribute to transient fibrosis formation and following this, they partially return to the quiescent stage (inactivation) in order to drive fibrosis regression and heart regeneration, as ablating *col1a2*-expressing fibroblasts impaired CM proliferation and scar resolution [110]. Thus, the fibrotic response is critical for scar formation (in regenerative and non-regenerative models) as well as for scar resolution (in the regenerative models only).

In addition to cardiac tissue activation, immune cell populations respond to heart injury by promoting inflammation. A transient delay of macrophage recruitment, using clodronate liposome (CL) injections one day prior to injury was sufficient to disrupt neovascularization, neutrophil clearance and heart regeneration following cardiac injury in zebrafish [108]. Neutrophils are also recruited to the injured area and cooperate with macrophages and monocytes to promote the onset and resolution of inflammation [108,111]. It was recently observed that upon macrophage depletion, neutrophil retention leads to unsolved fibrotic scars in zebrafish and comparison to the non-regenerative model medaka revealed that neutrophil clearance is delayed to the hearts with no regeneration capacity [103]. Recently, Hui et al. highlighted the role of zebrafish specialized regulatory T cells (T_reg_) which infiltrate the damaged heart and produce tissue-specific mitogens essential for robust regeneration [112]. Finally, studies in the Mexican cavefish that fail to regenerate their hearts when compared to their surface fish relatives were used to successfully identify quantitative trait loci linked to the ability of heart regeneration [113]. Therefore, comparisons between regenerative and non-regenerative models, studies of the role of the innate and adaptive immune system, cardiac tissue remodeling and cellular contributions in cardiac repair are important to elucidate the mechanism underlying heart regeneration and promote therapeutic strategies for post-injury responses. The field of regenerative medicine is now focusing on the dynamic crosstalk between immune cells and cardiac-derived stroma cells (reviewed in [114]). Parallel studies in regenerating organisms such as zebrafish and non-regenerating ones will surely unravel the underlying mechanism needed to be re-activated in adult mammals to allow regeneration.

## 6. Future Perspectives

In summary, zebrafish have emerged as a valuable tool in biomedical research and contributed to the deeper understanding of cardiac development and pathophysiology. Due to the efforts of forward genetic screens and manipulation, zebrafish research has been pivotal in unraveling major factors of cardiac function as well as the molecular pathways in which they are involved. The efforts that had been made out of the combination of population-based GWAS and functional analysis in zebrafish models shed light on uncharacterized mechanisms that give the knowledge to design new prognostic and therapeutic strategies. The growing toolbox of transgenic zebrafish lines, tissue-specific genetic manipulation, high-resolution imaging and high-throughput chemical screens will provide an excellent model in clinical and basic research to dissect the architecture of cardiac diseases.

## Figures and Tables

**Table 1:** An indicative table of GWAS-derived cardiomyopathy related genes studied in zebrafish.

Associated Human Disease	Gene (s)	Zebrafish Genotype	References
Atrial Fibrillation	*KCNIP1*	High atrial rate	[80]
Long QT Syndrome	*KCNH2*	Severe repolarization	[81]
Atrial Fibrillation	*PRRX1*	Atrial action potential duration	[82]
Dilated Cardiomyopathy	*HSPB7*	Cardiac fibrosis, cardiomegaly and sarcomeric abnormalities	[83]
Congenital Heart Defects	*PBX3*	Myocardial morphogenesis defects	[84]
Mitral Valve Prolapse	*LMCD1*, *TNS1*	Increased atrioventricular regurgitation, moderate reduction in cardiac looping	[86]
Atrioventricular Septal Defect	*NFATC1*	Cardiac looping defects and altered atrioventricular canal patterning	[87]
Heart Rate Variability	*GNG11*, *SYT10*, *RGS6, HCN4*, *NEO1*, *KIAA1755*	Sinoatrial pauses and arrests, cardiac edema and uncontrolled atrial contractions	[88]
Lipid Associated-Cardiomyopathy	*APOOP1*	Increased the LDL-C levels, increase in the average number of vascular plaques	[95]
Lipid Associated-Cardiomyopathy	*PLXND1*	Modulate angiogenesis, reduced capacity to store lipid in visceral adipose tissue	[96]
Congenital Cardiomyopathy	*KIF20A*	Relative tachycardia, red blood cells proximal to the atrium and cardiac edema	[97]
